# Nutritional Modulation of Metabolic–Endocrine Integration and Growth Performance in Tropical Dairy Heifers

**DOI:** 10.3390/ani16193053

**Published:** 2026-09-28

**Authors:** Andi Mushawwir, Ujang Hidayat Tanuwiria, Iin Susilawati, Nyimas Popi Indriani, Dwi Suharwanto, Fida Prawita, Rizal Lesmana, Salwa Iffat Zahida Arif

**Affiliations:** 1 Faculty of Animal Husbandry, Universitas Padjadjaran, Jl. Ir. Soekarno KM 21, Sumedang 45363, West Java, Indonesia; ujang.hidayat@unpad.ac.id (U.H.T.); iin.susilawati@unpad.ac.id (I.S.); nyimas.popi@unpad.ac.id (N.P.I.); dwi.suharwanto@unpad.ac.id (D.S.); 2 Centre for Breeding and Development of Artificial Insemination for Dairy Cattle (BPPIBTSP), Food Security and Livestock Agency of West Java, Bunikasih, Warungkondang, Cianjur 43261, West Java, Indonesia; medikvet.drhfida.dkpp@gmail.com (F.P.); rizallespaul@gmail.com (R.L.); salwaiffat24@gmail.com (S.I.Z.A.)

**Keywords:** blood biomarkers, energy nutrition, haematology, metabolic status, short-term response

## Abstract

This study evaluated short-term haematological, metabolic, endocrine, and growth responses to calcium-protected oil and cornmeal in tropical dairy heifers. We allocated 128 heifers to four dietary treatments (32 heifers per treatment): R1, 60% roughage + 40% concentrate; R2, R1 with 4% Ca-oil; R3, R1 with 4% cornmeal; and R4, R1 with 2% Ca-oil + 2% cornmeal. We assessed blood biomarkers at two monthly sampling points and analysed weekly growth longitudinally. We detected treatment differences in erythrocytes, haemoglobin, MCV, MCH, MCHC, RDW-CV, eosinophils, HDL cholesterol, total protein, and albumin (*p* < 0.05). We found no statistically detectable treatment effects for most leukocyte indices, energy metabolites, reproductive hormones, growth-related hormones, or cortisol. Body measurements increased over time (*p* < 0.001), and we detected a treatment × week interaction only for body length (*p* = 0.014). The combined diet was associated with the highest values for several erythrocyte and protein variables, but the design does not distinguish synergy from additive or dose effects. These results should therefore be interpreted as short-term associations requiring confirmation through fully specified repeated-measures analyses and longer-term functional outcomes.

## Simple Summary

Dairy heifers require adequate dietary energy to support growth and physiological development. This study compared four forage-concentrate diets containing calcium-protected oil, cornmeal, or a combination of both in 128 heifers. The supplemented diets were associated with differences in selected erythrocyte indices, HDL cholesterol, total protein, and albumin. In contrast, no statistically detectable treatment effects were observed for most measured metabolites and hormones. All groups grew during the study, and the treatment-by-week interaction was confined to body length. The findings describe short-term response patterns and do not establish reproductive benefits or a synergistic effect of the combined ingredients.

## 1. Introduction

The heifer phase influences subsequent growth and reproductive readiness. Short-term nutritional studies cannot determine age at puberty, ovulation, conception, or long-term reproductive performance; however, they can characterise early haematological, metabolic, and endocrine response patterns that may inform longer-term investigations [1,2,3,4,5,6,7].

Adequate energy provision is required for somatic growth, although increased energy density does not necessarily produce reproductive benefits. Studies in dairy heifers show that energy allowance and growth pattern can affect feed efficiency, body development, and metabolic indicators [8,9]. These observations support short-term multiparameter monitoring, provided that biomarker changes are not interpreted as direct evidence of accelerated reproductive maturation [10,11,12,13,14].

Different energy sources may support distinct metabolic pathways. Corn starch supplies fermentable substrate and glucogenic precursors, whereas calcium-protected oil increases dietary energy density while limiting disruption of ruminal fermentation. Evidence from young cattle indicates that dietary starch supply can influence fermentation, nutrient utilisation, and circulating metabolites [15,16,17,18,19,20,21].

Rumen-protected fat or calcium salts of fatty acids can increase dietary energy density, although responses depend on basal ration composition, physiological stage, and inclusion rate [22,23,24]. Combining cornmeal with protected fat could distribute energy supply between ruminal starch fermentation and post-ruminal fatty-acid delivery, potentially reducing reliance on a single substrate. In R4, however, each ingredient was supplied at half the dose used in the corresponding single-ingredient treatment; therefore, the comparison cannot establish synergy and is interpreted only as a comparison among four formulated diets [13,25,26].

Nutritional evaluation in heifers should not rely solely on glucose or body weight. Haematology provides information on oxygen-carrying capacity, erythropoiesis, and blood cell status, while the leukocyte differential can help assess immune stability and stress. Haematological reference ranges in cattle vary by age and physiological status, so interpretations should consider the population context and production phase [27,28,29]. At the same time, total protein, albumin, lipids, NEFA, BHBA, and metabolic hormones provide complementary information on nutrient availability, energy mobilisation, and homeostasis.

Energy status can influence ovarian activity, making reproductive biomarkers relevant to nutritional assessment. Anti-Müllerian hormone (AMH), a marker associated with the antral follicle population, can provide complementary information on ovarian reserve, although a short intervention cannot establish long-term reproductive capacity [30,31,32,33,34]. AMH and other reproductive hormones must also be interpreted in relation to developmental and ovarian stage [35,36,37,38].

This study tested whether four formulated diets produced distinct short-term response patterns within and across haematological, metabolic, endocrine, and growth domains. We hypothesised that the diet containing both Ca-oil and cornmeal would differ from the basal and single-ingredient diets in selected biomarkers. Because R4 contained half-doses of both ingredients, the study was not a 2 × 2 factorial experiment and was not designed to test synergy. The objective was to characterise short-term responses over two months rather than to infer long-term reproductive development.

## 2. Materials and Methods

### 2.1. Study Design

This study involved 128 FH-crossbred dairy heifers, with an average BCS of 3.25, allocated to four dietary treatments (32 animals per treatment) and housed in individual pens. We dewormed the animals before starting the dietary treatments. We measured the same animals at month 1 and month 2, yielding up to 256 repeated biomarker observations per variable, assuming no missing samples. At the start of the treatment, all heifers were 10 months old with an average body weight of 195.56 ± 6.34 kg. The treatments applied were: R1 = 60% forage + 40% concentrate; R2 = basal diet formulated with 4% calcium oil (Ca-oil); R3 = basal diet formulated with 4% corn flour; and R4 = basal diet formulated with 2% calcium oil + 2% corn flour. These four diet types were treated as categorical interventions; the study design was not factorial, and the researchers did not analyse interactions between calcium oil and corn flour. Researchers monitored each heifer longitudinally throughout the two-month sampling period.

Feeding was carried out twice daily (morning and afternoon), with a total ration of 25 kg (R1) supplemented according to the respective treatment protocols. Water containers were placed next to the feed troughs to provide access to drinking water throughout the day. The average temperature and humidity during the study were 27 °C and 75%, respectively.

### 2.2. Sampling and Laboratory Analysis

Blood samples were collected from the same heifers at months 1 and 2 via jugular venipuncture using a sterile 3 mL syringe and then transferred into two separate tubes. Blood collected in EDTA tubes was gently inverted and used for haematological analysis. In contrast, blood intended for serum biochemical and hormonal assays was placed in plain tubes (without anticoagulant) and allowed to clot at room temperature for 30–45 min. Both EDTA-anticoagulated blood and clotted blood were centrifuged at 3500 rpm for 10 min at 4 °C; plasma and serum were then carefully separated from the cellular fraction, aliquoted into labelled microtubes, and stored at −80 °C until analysis, avoiding repeated freeze–thaw cycles. Haematological measurements were performed on fresh whole blood containing EDTA as soon as possible after collection, and samples exhibiting visible hemolysis, lipemia, or insufficient volume were excluded from biochemical and endocrine analyses.

Haematology parameters were determined using a Mindray BC-5300 haematology analyser (manufactured by Shenzhen Mindray Bio-Medical Electronics Co., Ltd., Shenzhen, China). A tube containing whole blood was placed beneath the aspiration probe, and the instrument automatically aspirated 20 µL of the sample. The parameters included erythrocytes, haemoglobin, hematocrit, MCV, MCH, MCHC, RDW-CV, total leukocyte count and differential count, neutrophil–lymphocyte ratio, platelet count, and MPV.

We analysed serum concentrations of glucose, NEFA, BHBA, triglycerides, total cholesterol, HDL-C, LDL-C, total protein, albumin, BUN, creatinine, and lactate using commercial diagnostic kits from Randox Laboratories Ltd. (Crumlin, UK) according to the manufacturer’s instructions. We performed analyses spectrophotometrically using enzymatic colourimetric or kinetic methods at wavelengths appropriate for each analyte: 340 nm for BHBA and BUN; 500–600 nm for energy and lipid metabolites; 540–560 nm for total protein; 600–630 nm for albumin; and 490–550 nm for creatinine and lactate. We calculated globulin concentration by subtracting albumin from total protein. We analysed each sample in duplicate using blanks, calibrators, and low- and high-concentration controls; we accepted results if the intra-assay coefficient of variation was ≤10%, controls fell within established ranges, and linearity and recovery in the bovine serum matrix were within 80–120%.

Serum concentrations of oestradiol-17β, progesterone, LH, FSH, AMH, prolactin, leptin, GH, IGF-1, insulin, T3, T4, and cortisol were analysed using bovine-specific ELISA kits from MyBioSource Inc. (San Diego, CA, USA) according to the manufacturer’s instructions. Peptide and protein hormones were analysed using the sandwich ELISA principle, whereas steroid and thyroid hormones were analysed using competitive ELISA; absorbance was measured at 450 nm with reference correction at 570 or 620–630 nm, and concentrations were calculated from a four-parameter logistic regression standard curve. All samples were analysed in duplicate alongside blanks, standards, and internal controls, and were accepted if the intra-assay and inter-assay coefficients of variation were ≤10% and ≤15%, respectively. Kit performance in bovine serum was verified through linearity of dilution, parallelism, and spike-and-recovery assays, with an acceptable recovery range of 80–120%.

### 2.3. Statistical Analysis

For haematological, metabolic, endocrine, and stress biomarkers, the heifer was the longitudinal observational unit. The dataset comprised 128 heifers (32 per treatment) measured at two monthly time points. The originally submitted analysis used each heifer’s two-month mean in a one-way ANOVA with diet as the fixed factor, followed by Tukey’s HSD test when appropriate.

Weekly body weight, body length, chest circumference, and withers height were analysed using linear mixed-effects models with treatment, week, and treatment × week as fixed effects and heifer as a random effect. Week was treated as a categorical repeated factor. The final report should additionally state the residual covariance structure, estimation method, denominator degrees-of-freedom method, random-effects structure, baseline adjustment, model diagnostics, and multiplicity-adjustment procedure. We calculated ADG as the change from week 0 to week 7 divided by the exact observation interval; we recommend a sensitivity analysis based on the individual slope across all weekly weights. We calculated associations between ADG and IGF-1, NEFA, insulin, and glucose after adjustment for treatment, with 95% confidence intervals and *p*-values adjusted for multiplicity. We calculated heatmaps separately for each biomarker from the eight treatment × month means, using the mean and standard deviation across those eight cells; they are descriptive and do not represent individual variability or inferential evidence.

All analyses were performed using R version 4.6.1 (R Foundation for Statistical Computing, Vienna, Austria). Mixed models with residual covariance structures were analysed using the nlme package (version 3.1-171), while additional mixed-effects models were analysed using lme4 (version 2.0-6) and lmerTest (version 3.2-1). We implemented the Kenward–Roger approach using the pbkrtest package (version 0.5.5), and calculated Satterthwaite degrees of freedom using lmerTest (version 3.2-1). We estimated marginal means, confidence intervals, Tukey pairwise comparisons, and simple effects analyses using emmeans (version 2.0.4). Model diagnostics and goodness-of-fit evaluations were performed using the performance package (version 0.18.2), while effect sizes were calculated using effectsize (version 1.0.3). Where necessary, we conducted sensitivity analyses for missing data using the mice package (version 3-19.0). Heatmaps were generated using pheatmap (version 1.0.13), while diagnostic and results plots were created using ggplot2 (version 4.0.3). False discovery rate adjustment was performed using the p.adjust function with the Benjamini–Hochberg method from the base stats package (version 4.6.1).

## 3. Results

### 3.1. Haematological Response

Treatment differences were concentrated in selected erythrocyte measures (Table 1). R4 had the highest values for several erythrocyte-related variables, including erythrocytes and haemoglobin, while treatment effects were also detected for MCV, MCH, MCHC, and RDW-CV (*p* = 0.001, 0.003, 0.004, 0.018, 0.014, and 0.037, respectively). Haematocrit showed no statistically detectable treatment effect; this may reflect the joint influence of erythrocyte number, cell volume, and plasma volume, but plasma-volume data were not available. We found no statistically detectable treatment effects for most leukocyte or platelet measures. Eosinophils differed among diets (*p* = 0.038), although this isolated finding requires cautious interpretation after multiplicity correction.

### 3.2. Energy and Protein Metabolism

Treatment differences were detected for HDL cholesterol, total protein, and albumin (Table 2). In contrast, we found no statistically detectable treatment effects for glucose, NEFA, BHBA, triglycerides, total cholesterol, LDL cholesterol, BUN, creatinine, lactate, globulin, or the glucose:insulin ratio. Total protein was highest in R4, with R2 and R3 intermediate; because the diets were categorical rather than ordered doses, this pattern was not described as a gradual trend. The concurrent changes in albumin but not globulin are compatible with a difference in the albumin component of circulating protein. However, hepatic synthesis was not measured, and no mechanistic conclusion can be drawn.

### 3.3. Reproductive Hormones

No statistically detectable treatment effects were found for ooestradiol-17β, progesterone, LH, FSH, AMH, prolactin, or leptin (Table 3). This result does not demonstrate endocrine equivalence or stability, particularly because hormone concentrations depend on sampling time and ovarian stage.

Accordingly, the available analysis did not detect consistent diet-related differences in the measured reproductive hormones. Because the submitted methods did not fully characterise ovarian stage and pulsatile hormone secretion, these findings are limited to the concentrations measured at the two sampling points and do not support conclusions about long-term reproductive development.

### 3.4. Growth–Anabolic and Stress Axes

No statistically detectable treatment effects were found for GH, IGF-1, insulin, T3, T4, or cortisol (*p* > 0.05; Table 4). These non-significant findings do not demonstrate physiological equivalence among diets. They indicate only that the analysis used did not detect treatment differences at the two sampling points.

### 3.5. Multivariate Response Visualisation

We utilised Z-score-based heatmaps to examine the relative structure of each biomarker’s response across treatment combinations and observation months (Figure 1, Figure 2, Figure 3 and Figure 4). This visualisation complements univariate results by exposing temporal heterogeneity that may be hidden when two months are summarised as a single mean per heifer. Since Z-scores indicate a value’s position within the biomarker distribution, red or blue colours do not necessarily imply better or worse outcomes. In haematology, standardised Z-scores uncovered a more intricate temporal and treatment pattern than mean-based comparisons (Figure 1). R1 generally exhibited negative Z-scores for most erythrocytic parameters, especially in the first month, with several parameters increasing in the second month.

Conversely, R4 mainly showed positive Z-scores for various erythrocytic indicators, including erythrocytes, haemoglobin, MCH, MCHC, RDW-CV, and MCV in one or both observation months. This aligns with the univariate findings, which indicated that the dietary response mainly affected the erythrocytic compartment. Leukocyte responses were more heterogeneous, with the neutrophil: lymphocyte ratio relatively high in R1-M1 and R3-M2 but lower in other treatment–month combinations. WBC and neutrophils also fluctuated between months without a consistent pattern across R1 to R4. Overall, the R4 haematological pattern is more noticeable in erythrocytic parameters than in leukocytes and exhibits temporal variations that the two-month average analysis does not fully capture.

The energy metabolism heatmap showed greater heterogeneity in responses than the haematology domain (Figure 2). Negative Z-scores characterised R1-M1 for several indicators, including glucose, HDL cholesterol, total protein, albumin, and globulin, while several parameters increased in R1-M2. R4 showed a different profile between the first and second months. Total protein and albumin showed positive Z-scores, particularly in R4, consistent with the univariate analysis. Parameters directly related to energy mobilisation did not move in parallel. NEFA was relatively high in R3-M1 and R4-M1 but decreased in R4-M2, while BHBA also showed a directional change between the first and second months in some treatments. Glucose in R4 was above the mean in both months, but this pattern did not produce significant treatment differences in the univariate analysis. Therefore, the heatmap suggests that metabolic adaptation is parameter- and time-specific, rather than a uniform shift across all metabolites toward higher or lower values.

The reproductive hormone Z-score profiles showed fairly clear intermonthly fluctuations but did not yield a consistent single-treatment pattern (Figure 3). R2 showed positive Z-scores for oestradiol-17β in the first month and LH and FSH in the second month. Conversely, some of these hormones had negative Z-scores in other treatment–month combinations.

R3-M2 showed positive Z-scores for progesterone and leptin, while R4 showed different patterns between the first and second months for AMH, oestradiol, progesterone, leptin, and prolactin. This variability in response direction is consistent with the absence of significant differences between treatments in the reproductive hormone panel. In other words, the heatmap indicates that the hormonal variations reflected temporal dynamics and heterogeneity among hormones rather than coordinated shifts in the overall reproductive axis in response to a specific diet.

The growth hormone–anabolic heatmap also shows a pattern that depends strongly on the month of observation (Figure 4). Insulin shows relatively high Z-scores in R1-M1 and R4-M1, but lower in some other combinations.

GH exhibits relatively high values in R1-M2 and R4, while IGF-1 exhibits positive Z-scores, particularly in R4-M2. T3 increases relatively strongly in R4-M1, while T4 exhibits the highest Z-score in R4-M2. Conversely, cortisol reaches relatively high Z-scores in R3-M1 and lower in R4. However, because there are no significant differences between treatments for GH, IGF-1, insulin, T3, T4, or cortisol, these colour patterns should be viewed as relative structure within the dataset, not evidence of a significant treatment effect. The heatmap primarily reveals that these hormones do not move synchronously and that the first-month response is not always maintained in the second month.

When all four biomarker groups are considered together, a pattern emerges that the response to the nutritional strategy is hierarchical and domain-specific. Statistical evidence is clearest in the erythrocytic compartment and circulating proteins and lipoproteins, while key energy metabolites and the endocrine axis show greater stability. R4 produced relatively prominent profiles for erythrocytes, haemoglobin, and total protein, but consistent increases in all growth or reproductive hormones did not accompany this superiority. The heatmap further demonstrates that treatment effects cannot be completely separated from the temporal dimension. Several biomarkers exhibited substantial Z-score shifts from the first to the second month within the same treatment. Overall, the results indicate that physiological adaptation to the diet is a multidimensional, dynamic response, with haematological–metabolic changes more pronounced than endocrine changes over the two months of observation.

### 3.6. Longitudinal Growth and Growth–Metabolic Integration

All growth indicators changed significantly by week of observation (*p* < 0.001; Figure 5 and Figure 6), confirming progressive somatic growth throughout the study period. Treatment had no significant main effect on body weight, body length, chest circumference, or withers height. However, we detected a treatment × week interaction for body length (*p* = 0.014), indicating that body length trajectories differed between feeding strategies. We found no such interaction for body weight (*p* = 0.306), chest circumference (*p* = 0.992), or withers height (*p* = 0.985).

Exploratory correlation analysis suggested several candidate associations, but no edge remained significant after false-discovery-rate correction (q < 0.05). The network therefore provides no robust evidence of physiological modules or causal integration and should be regarded solely as hypothesis-generating material for confirmation in an independent, adequately powered dataset (Figure 7).

## 4. Discussion

The main finding of this dataset is that energy source manipulation produces a more pronounced response in haematological compartments and circulating proteins than in classical energy metabolites or the reproductive endocrine axis. R2 and especially R4 increase red blood cells and haemoglobin, accompanied by changes in MCV, MCH, MCHC, and RDW-CV. Physiologically, this pattern reflects changes in oxygen-transport capacity and red blood cell characteristics but does not imply direct stimulation of erythropoiesis. Haematological interpretation in cattle must consider age, physiological status, hydration, and population biological variation [27,28,29]. Because this study did not measure reticulocytes, Fe, vitamin B12/folate, or erythropoietin, the erythrocytic responses are better described as changes in haematological phenotype rather than evidence of a specific hematopoietic mechanism.

The absence of statistically detectable treatment effects for WBC, neutrophils, lymphocytes, the neutrophil: lymphocyte ratio, and cortisol should not be interpreted as evidence of equivalence or systemic stability. The isolated eosinophil difference in R2 may represent random variation, particularly after accounting for multiple testing, and its biological significance remains unclear.

The analysis did not detect treatment differences in glucose, NEFA, BHBA, triglycerides, or the glucose: insulin ratio. These findings do not establish metabolic stability or equivalence, because effect estimates, confidence intervals, repeated-measures modelling, and multiplicity control are required to judge the magnitude and precision of possible differences [5,6,8,9].

Interpret responses to cornmeal in relation to starch availability and ruminal fermentation. Dietary starch can influence fermentation, nutrient utilisation, and circulating metabolites, but this study tested only one corn form and did not measure ruminal fermentation or individual nutrient intake [15,16,17,18,19,20,21]. Accordingly, the absence of a detectable glucose difference does not establish either efficacy or inefficacy of cornmeal.

The higher HDL cholesterol values in supplemented groups may reflect differences in lipid transport. In contrast, higher total protein and albumin without a detectable globulin difference may indicate that the response occurred primarily in the albumin fraction. Nevertheless, hepatic synthetic rate, immunoglobulin fractions, nutrient intake, and detailed lipoprotein profiles were not measured; therefore, altered hepatic synthesis or immune protein mobilisation cannot be established [22,23,24].

R4 combined 2% Ca-oil with 2% cornmeal and showed the highest values for several erythrocyte and circulating-protein variables. A plausible explanation is that fermentable starch and protected lipid supplied energy through partly distinct routes. However, R4 contained half the dose of each ingredient used in R2 and R3, and the study was not factorial. The observed pattern therefore cannot distinguish synergy from additive effects, dose reduction, or differences in dietary energy density.

In the reproductive axis, unchanged levels of oestradiol, progesterone, LH, FSH, AMH, prolactin, and leptin indicate that the four diets did not produce consistent endocrine shifts over the observation period. This is not surprising, as puberty in heifers results from integrating physiological age, body weight, adiposity, growth, and metabolic signals [1,3]. Experimental evidence also suggests early nutritional carryover to reproductive tract development and follicle population, although endocrine changes are not always persistent [39]. Nutritional experiments show that leptin and LH can change with the plane of nutrition and developmental rate [6]. In contrast, other controlled studies show that nutritional influences on pubertal timing do not always translate into changes in adult reproductive function [36]. Recent studies have also shown that accelerated puberty through nutritional programs does not necessarily decrease ovarian responsiveness or oocyte competence, despite molecular responses in oocytes [10].

AMH stability, in particular, deserves separate discussion. AMH is a relatively useful biomarker for describing ovarian reserve and antral follicle population in cattle [30,31,40]. Researchers have evaluated a single AMH measurement for selecting donor ovum pick-ups [32], while longitudinal studies have shown patterns of AMH change throughout development in dairy cattle [33]. Researchers have also studied the relationship between AMH and reproductivity in Bos indicus [34]. However, the developmental environment can influence ovarian reserve: maternal status and prenatal environmental exposures have been reported to be associated with AMH or ovarian reserve in offspring [35,37]. Non-nutritional factors, such as health interventions, can also be evaluated against oestrous cycle parameters and AMH in heifers [38]. Therefore, the absence of changes in AMH in this study is more accurately interpreted as no evidence of changes in ovarian reserve within a two-month window, rather than evidence that nutrition has no long-term reproductive effects.

Z-score heatmaps enhance interpretation by highlighting temporal heterogeneity. Some biomarkers change direction between months 1 and 2 of the same treatment, so analyses using only two-month averages may mask treatment × month interactions. Biologically, these temporal patterns matter because nutritional responses in heifers vary with age and developmental stage. Longitudinal monitoring has been used to elucidate heifer metabolic adaptations to different nutritional programs [7,9], and recent reproductive research also emphasises the importance of linking age at puberty to ovarian traits longitudinally [10]. Therefore, for manuscripts, analyse raw data with linear mixed repeated-measures models that preserve heifer identity, include treatment, month, and treatment × month as fixed effects, and report effect sizes and their uncertainties.

Longitudinal growth analysis broadens interpretation by showing that time drives changes in body weight and size. Heifer growth should ideally be assessed as a trajectory, rather than simply final weight, because postweaning nutritional targets should align with ADG, frame size, growth composition, and reproductive readiness [41]. Population studies also indicate that nutrient supply, health, environment, and management influence ADG [42]. At the same time, long-term monitoring suggests that weight advantages can persist without parallel changes in overall body size or feed efficiency [43]. In this dataset, the significant treatment × week effect on body length but not on weight, chest circumference, or withers height suggests a trait-specific growth response.

The positive ADG–IGF-1 relationship provides physiological support for the somatotropic axis’s involvement in individual growth variation. IGF-1 is a key mediator of anabolic responses to nutrient availability; in dairy heifer calves, higher levels of nutrition can alter the concentrations of IGF-1 and its binding proteins [44], while differences in pre- and post-weaning nutrient supply affect growth, efficiency, glucose, insulin, IGF-1, and developmental indicators [5,45,46]. The finding that ADG does not correlate with glucose or insulin but does correlate with IGF-1 suggests that cumulative growth signals may be more informative than metabolites, which are strongly influenced by feeding time and sampling [47]. Metabolomics studies also indicate that nutrient supply shapes responses across energy, protein, and hepatic function pathways [45].

The nominal ADG-NEFA and ADG-IGF-1 correlations may generate hypotheses about energy partitioning and somatotropic signalling, but they do not establish mechanisms or clinical energy balance. Because none of the network edges remained significant after FDR correction, the analysis provides no robust evidence of integrated physiological modules. It should be confirmed using treatment-adjusted models in an independent dataset.

Overall, the study identifies short-term differences in selected haematological and circulating-protein measures among four diets. Interpretation is limited by incomplete reporting of animal characteristics, diet composition, intake, pre-analytical procedures, ovarian stage, assay performance, and the need for repeated-measures reanalysis with multiplicity control. These limitations preclude claims of endocrine stability, reproductive benefit, or synergy and should guide a confirmatory study with complete intake, growth, and reproductive outcomes.

## 5. Conclusions

Across the four diets, treatment differences were detected in selected erythrocyte indices, HDL cholesterol, total protein, and albumin, while most measured metabolites and hormones showed no statistically detectable effects. All groups grew, but the treatment × week interaction was limited to body length. R4 showed the highest values for several erythrocyte and protein measures; however, the design cannot distinguish synergistic, additive, dose, or energy-density effects. The findings are short-term and require confirmation after repeated-measures reanalysis and in longer studies with functional reproductive outcomes.

## Figures and Tables

**Figure 1:**
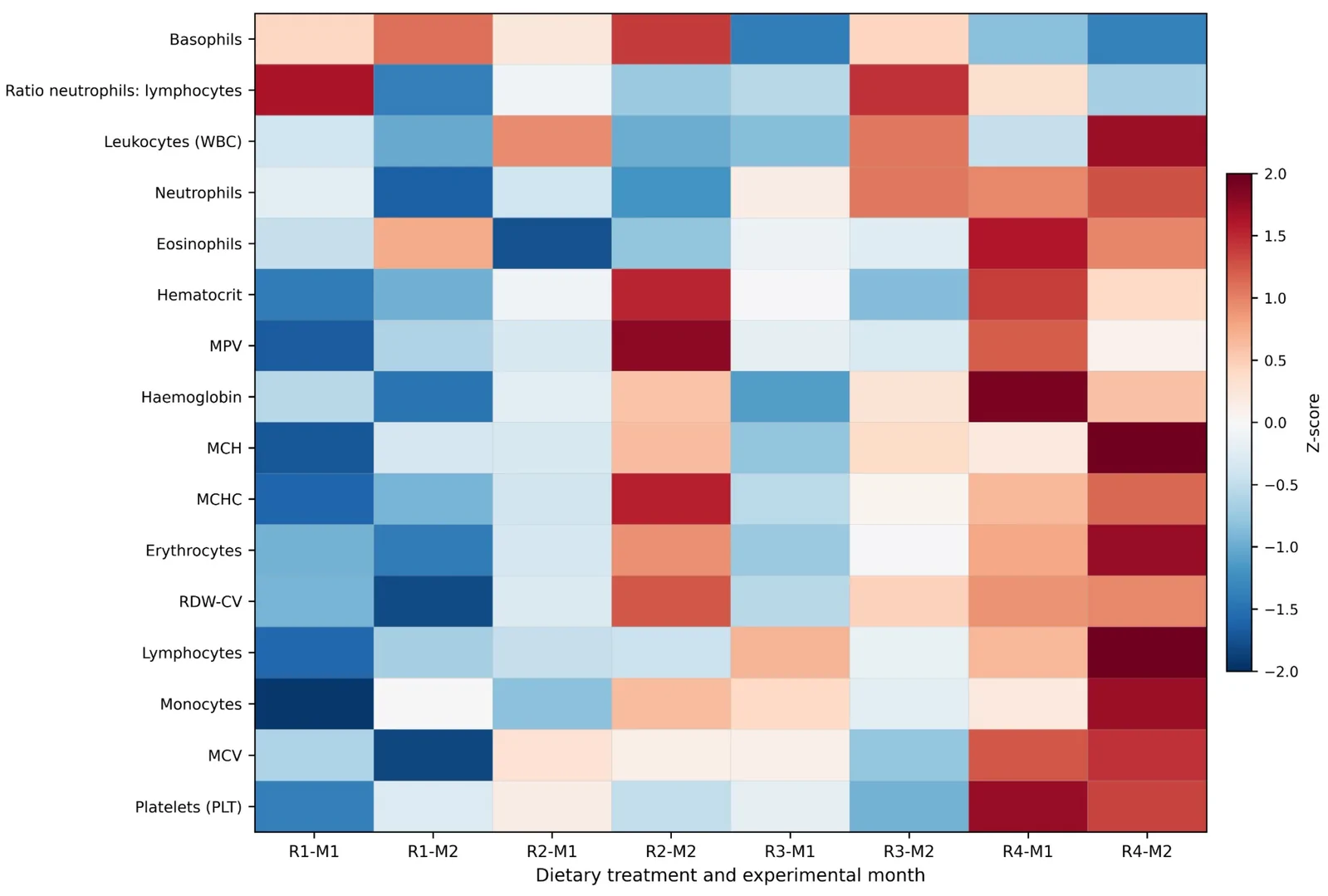
Heatmap of Z-scores for the haematological profile by ration treatment and observation month.

**Figure 2:**
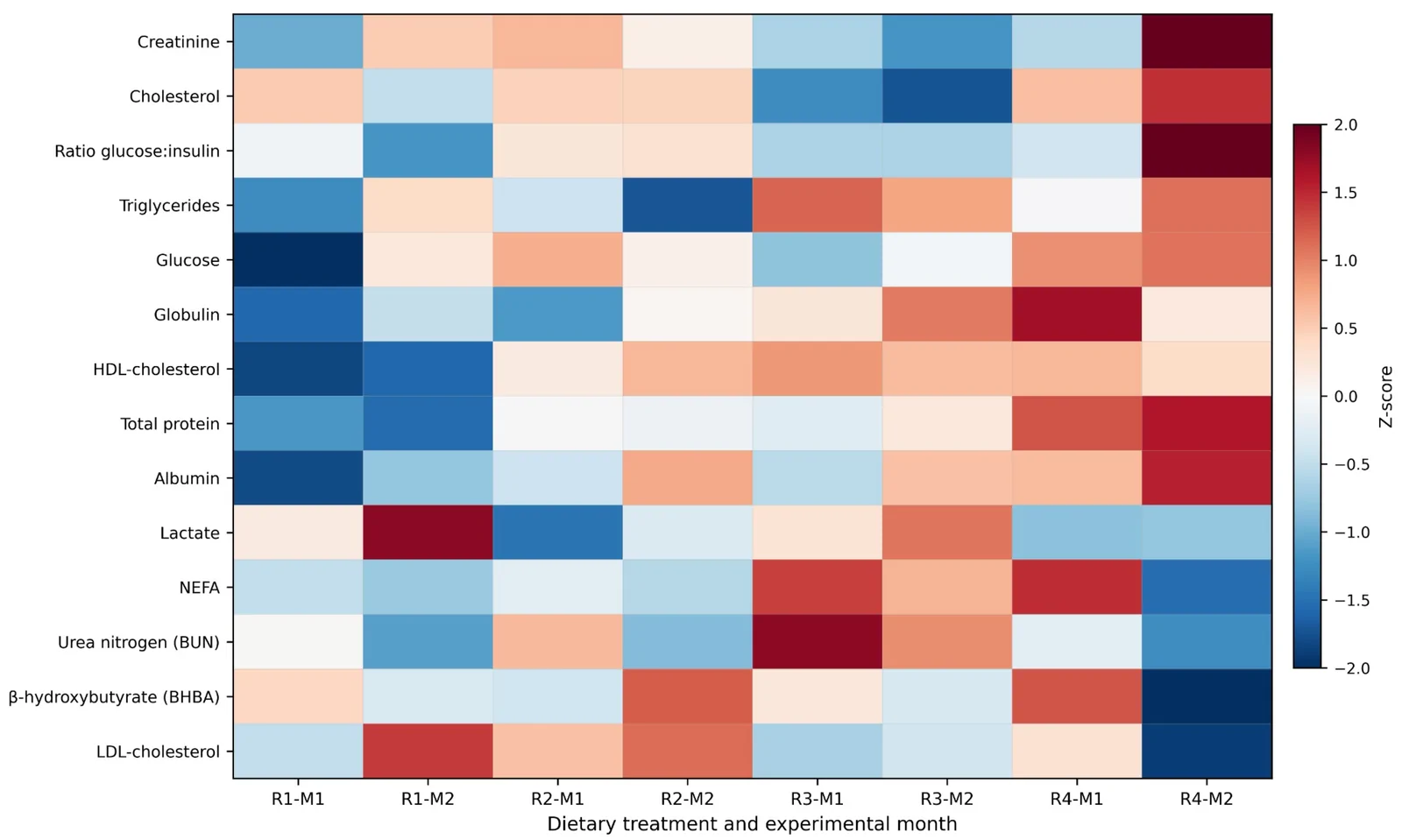
Heatmap Z-score of energy metabolism profile according to ration treatment and observation month.

**Figure 3:**
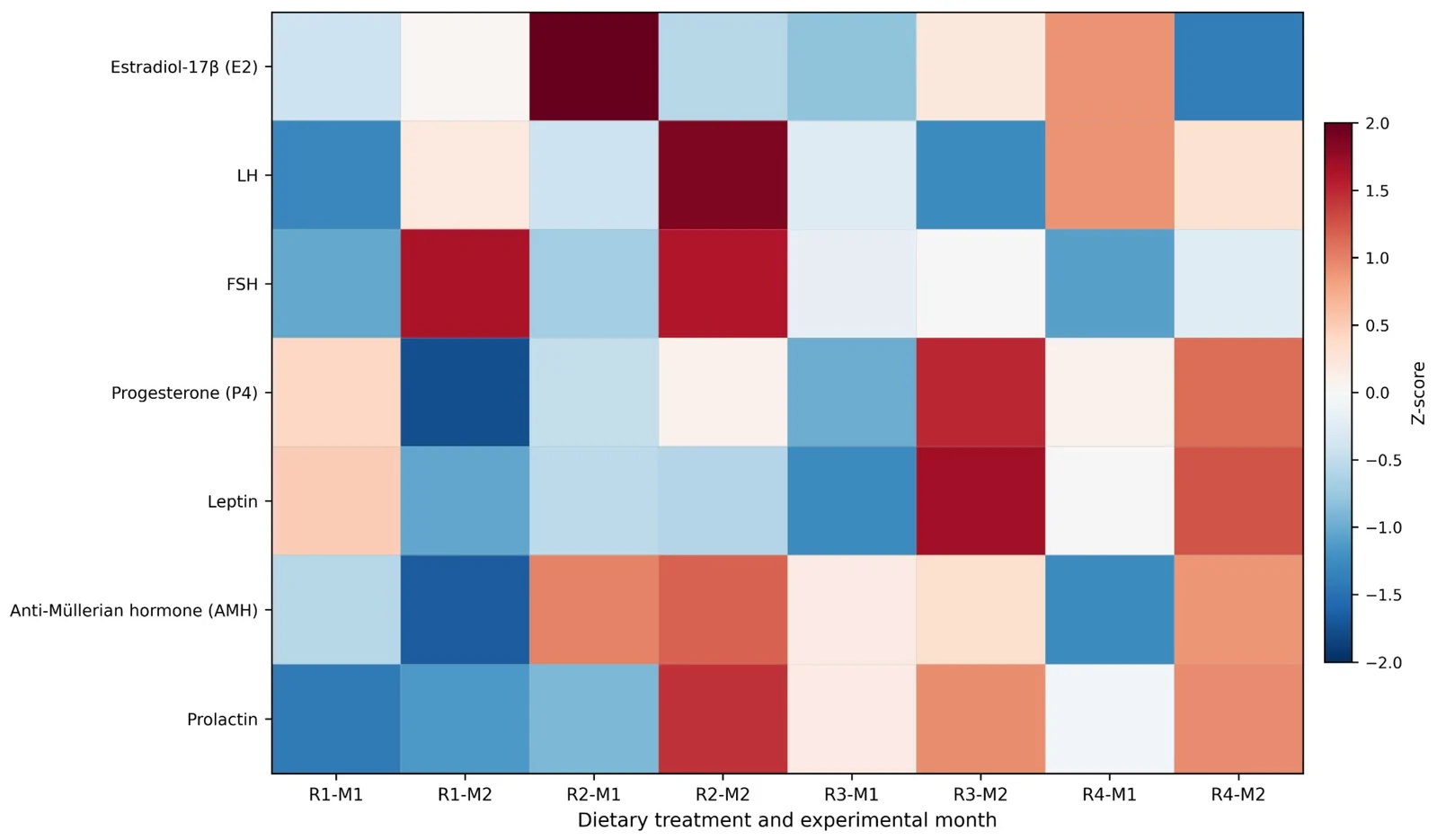
Heatmap of Z-scores of Reproductive hormones by ration treatment and observation month.

**Figure 4:**
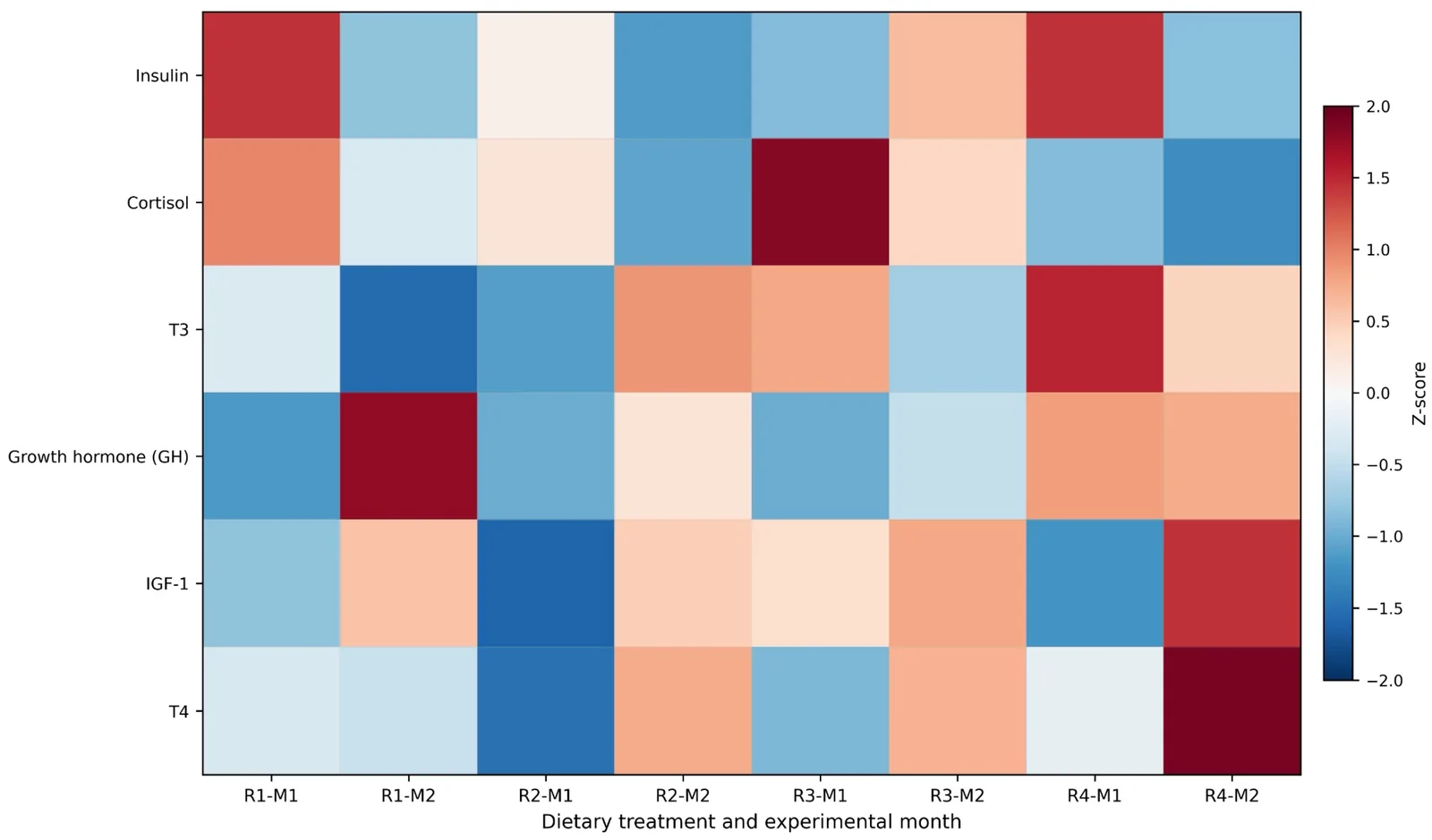
Heatmap of Z-scores of growth hormone–anabolic and cortisol according to ration treatment and month of observation.

**Figure 5:**
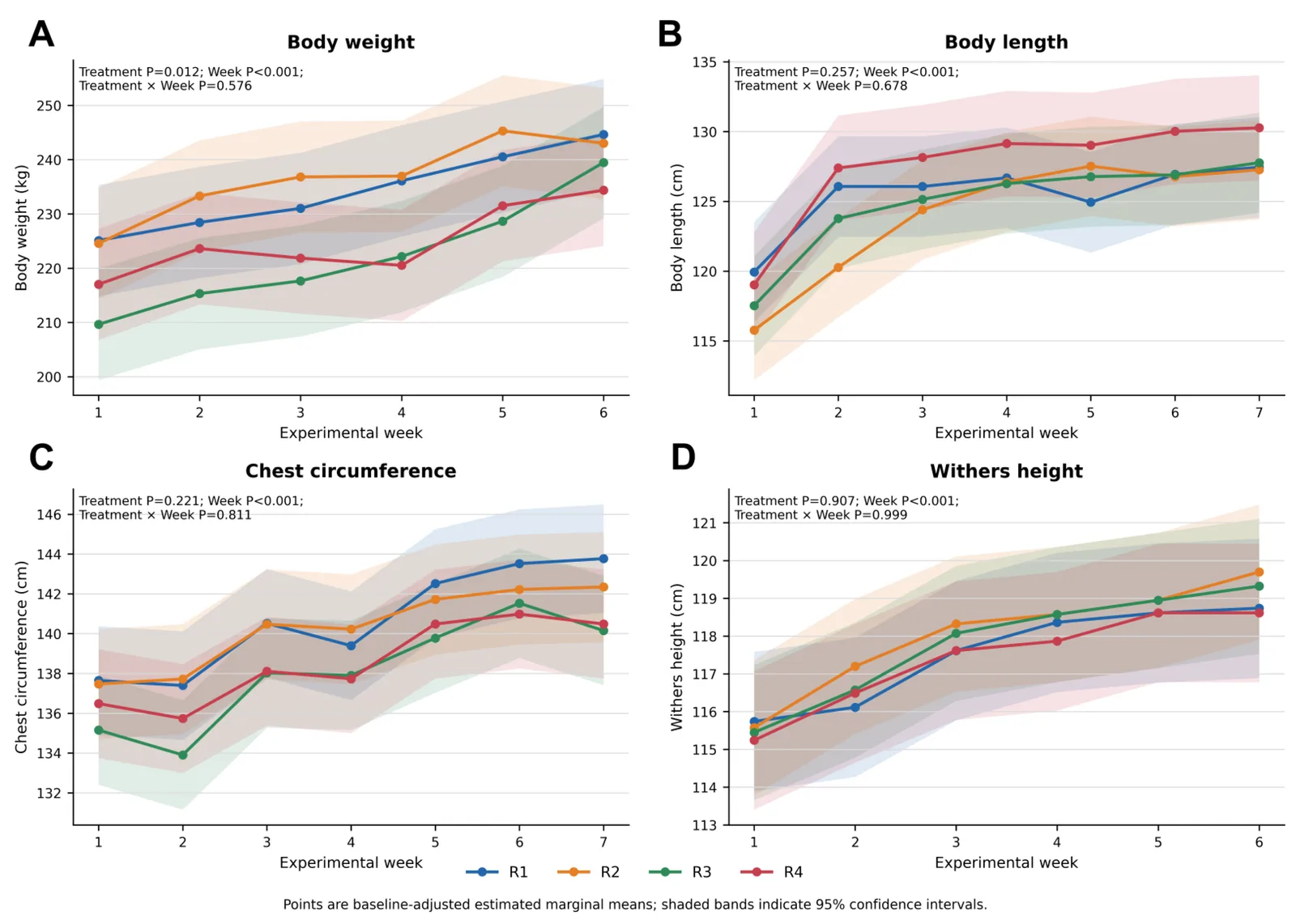
Longitudinal growth trajectories of tropical dairy heifers receiving four dietary treatments. (**A**) body weight, (**B**) body length, (**C**) chest circumference, and (**D**) withers height across the observation period. Lines represent treatment-specific trajectories; Longitudinal inference was based on a linear mixed model including treatment, week, and treatment × week, with heifer as a random effect.

**Figure 6:**
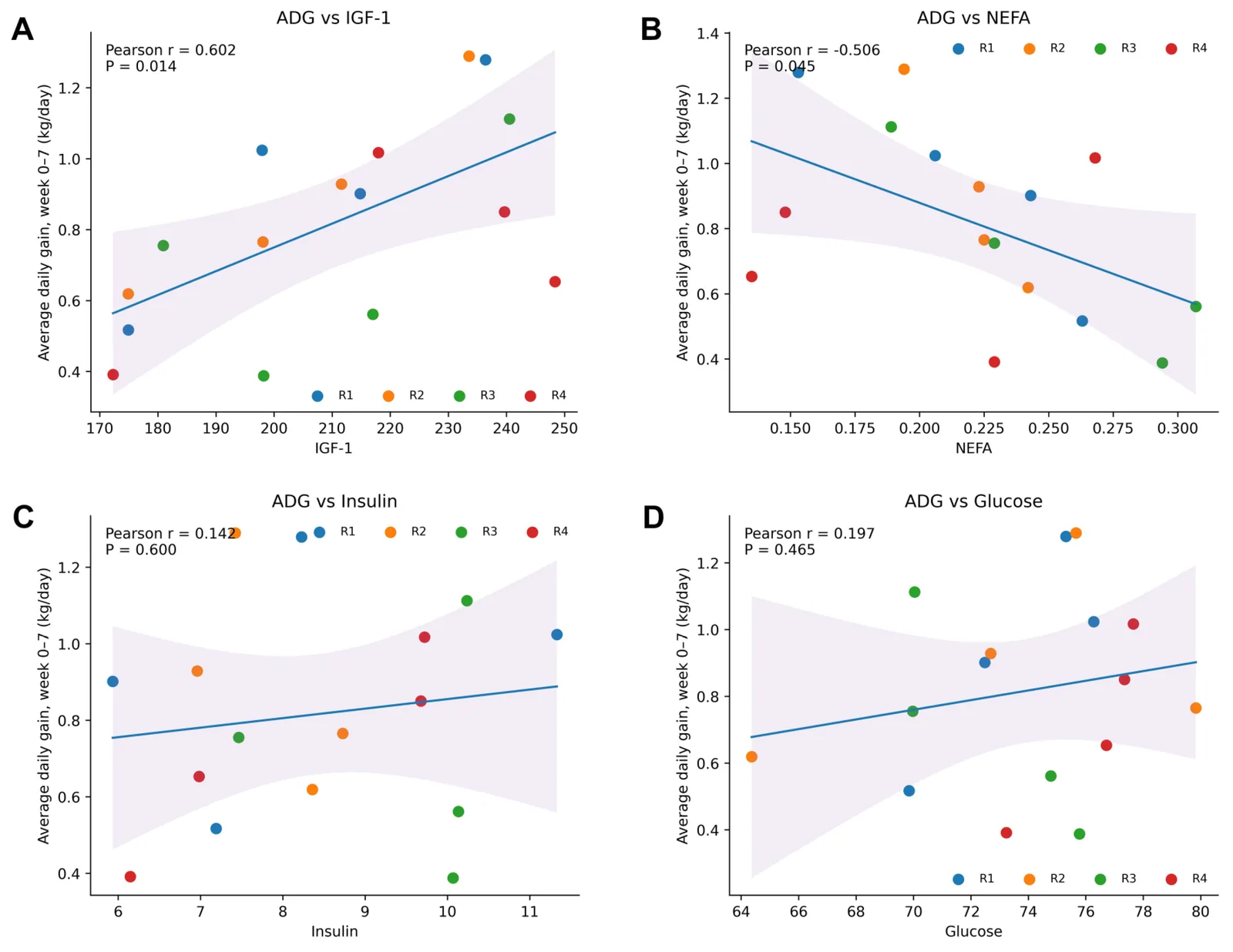
Associations between average daily gain (ADG) and selected metabolic–anabolic biomarkers in tropical dairy heifers. (**A**) ADG versus IGF-1, (**B**) ADG versus NEFA, (**C**) ADG versus insulin, and (**D**) ADG versus glucose. Pearson correlation coefficients and *p*-values are shown in the respective panels.

**Figure 7:**
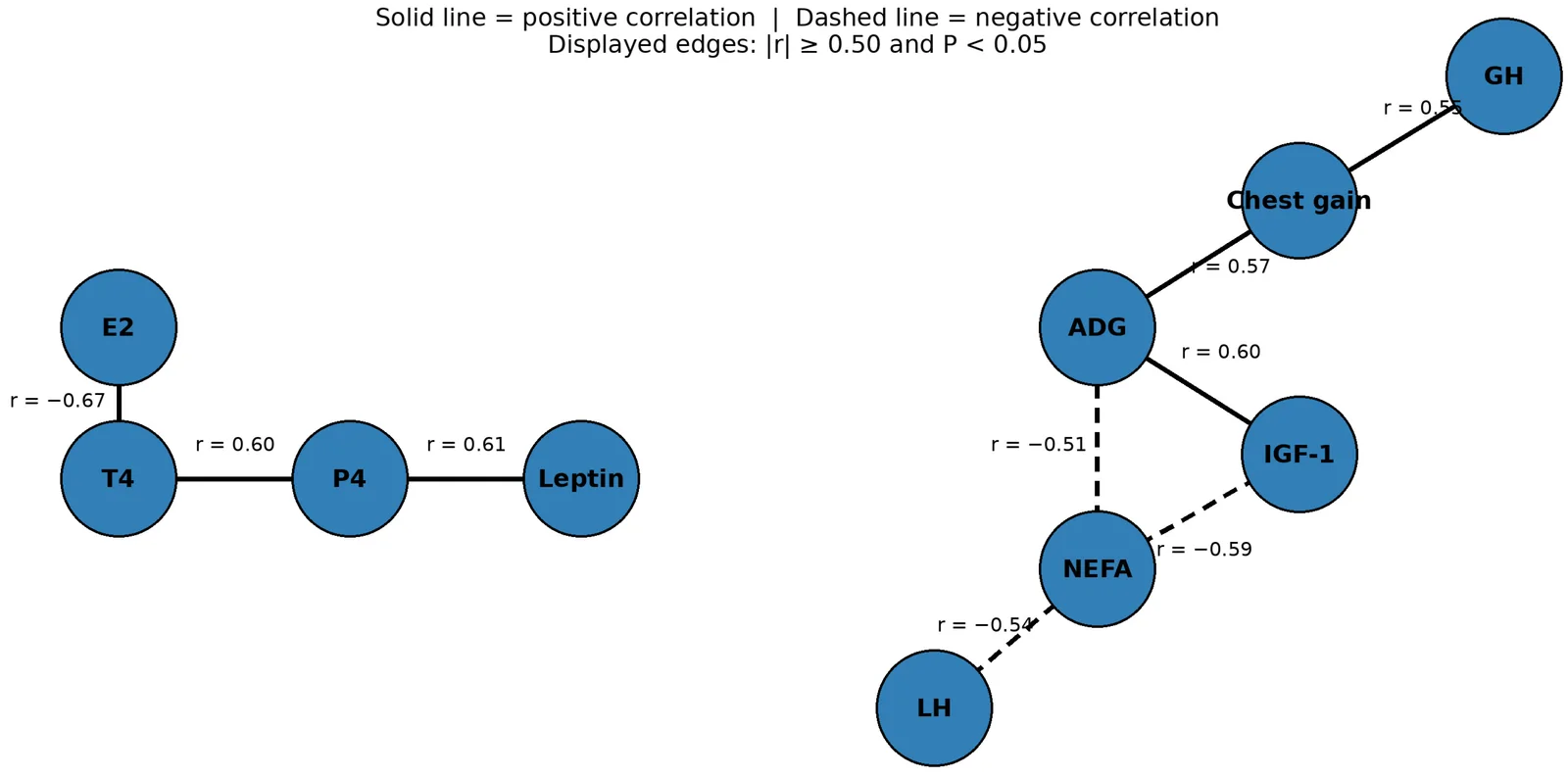
Exploratory integrated physiological response network linking growth, energy metabolism, and endocrine variables in tropical dairy heifers. Solid edges indicate positive correlations and dashed edges indicate negative correlations. Only associations with |r| ≥ 0.50 and *p* < 0.05 are displayed. ADG, average daily gain; GH, growth hormone; IGF-1, insulin-like growth factor-1; NEFA, non-esterified fatty acid; LH, luteinizing hormone; E2, oestradiol-17β; P4, progesterone.

**Table 1:** Haematological profile of dairy heifers.

Parameter	Unit	R1	R2	R3	R4	*p*
Erythrocytes	10^6^/µL	7.29 ± 0.06 b	7.72 ± 0.11 a	7.52 ± 0.26 b	8.01 ± 0.27 a	0.001
Hemoglobin	g/dL	11.14 ± 0.38 b	11.77 ± 0.29	11.45 ± 0.29 b	12.33 ± 0.43 a	0.003
Hematocrit	%	33.49 ± 0.21 a	35.47 ± 1.58 a	34.25 ± 1.65 a	35.64 ± 0.78 a	0.081
MCV	fL	46.83 ± 0.75 b	49.74 ± 2.11 a	48.63 ± 1.38 b	52.04 ± 1.77 a	0.004
MCH	pg	15.38 ± 0.73 b	16.32 ± 0.62 a	16.03 ± 0.40 a	17.06 ± 0.70 a	0.018
MCHC	g/dL	34.11 ± 0.91 b	35.88 ± 0.99 a	35.09 ± 0.76 a	36.18 ± 0.44 a	0.014
RDW-CV	%	18.67 ± 0.81 b	20.58 ± 1.26 a	20.04 ± 1.16 a	21.05 ± 0.88 a	0.037
Leukocytes (WBC)	10^3^/µL	9.36 ± 1.03 a	9.62 ± 1.03 a	9.67 ± 1.06 a	9.87 ± 0.30 a	0.886
Neutrophils	% WBC	37.53 ± 2.29 a	37.89 ± 2.71 a	41.63 ± 1.11 a	42.99 ± 4.71 a	0.059
Lymphocytes	% WBC	53.02 ± 1.53 a	55.45 ± 5.90 a	57.98 ± 5.64 a	61.70 ± 2.76 a	0.081
Monocytes	% WBC	5.00 ± 0.27 a	5.31 ± 0.57 a	5.37 ± 0.32 a	5.68 ± 0.25 a	0.142
Eosinophils	% WBC	1.66 ± 0.05 a	1.40 ± 0.14 b	1.60 ± 0.33 a	1.88 ± 0.17 a	0.038
Basophils	% WBC	0.57 ± 0.11 a	0.57 ± 0.20 a	0.53 ± 0.09 a	0.51 ± 0.07 a	0.877
RaNeutrLymphocytes: lymphocytes	ratio	0.71 ± 0.06 a	0.69 ± 0.07 a	0.73 ± 0.08 a	0.70 ± 0.10 a	0.934
Platelets (PLT)	10^3^/µL	415.83 ± 37.13 a	430.81 ± 28.01 a	421.83 ± 17.23 a	471.24 ± 23.10 a	0.056
MPV	fL	8.18 ± 0.22 a	8.74 ± 0.53 a	8.44 ± 0.77 a	8.71 ± 0.78 a	0.558

Values are the mean ± SD of the two-month average per heifer (n = 32 heifers/treatment; 128 heifers in total). Different letters in the same row indicate differences in Tukey HSD (*p* < 0.05).

**Table 2:** Energy and protein metabolism profile of dairy heifers.

Parameter	Unit	R1	R2	R3	R4	*p*
Glucose	mg/dL	69.75 ± 1.87 a	74.13 ± 3.82 a	71.47 ± 3.06 a	75.97 ± 3.63 a	0.072
NEFA	mmol/L	0.22 ± 0.04 a	0.23 ± 0.03 a	0.26 ± 0.03 a	0.24 ± 0.05 a	0.437
β-hydroxybutyrate (BHBA)	mmol/L	0.59 ± 0.04 a	0.61 ± 0.06 a	0.58 ± 0.09 a	0.55 ± 0.02 a	0.643
Triglycerides	mg/dL	18.72 ± 4.66 a	17.58 ± 1.01 a	21.31 ± 1.89 a	20.52 ± 2.00 a	0.260
Cholesterol	mg/dL	117.60 ± 4.98 a	120.41 ± 7.17 a	107.85 ± 13.38 a	124.18 ± 7.59 a	0.110
HDL cholesterol	mg/dL	62.62 ± 6.29 b	71.98 ± 4.42 a	73.47 ± 4.11 a	72.41 ± 3.56 a	0.023
LDL cholesterol	mg/dL	35.92 ± 2.52 a	37.19 ± 4.49 a	32.88 ± 4.08 a	31.98 ± 5.01 a	0.284
Total protein	g/dL	6.71 ± 0.10 c	7.07 ± 0.30 b	7.08 ± 0.14 b	7.49 ± 0.07 a	<0.001
Albumin	g/dL	3.29 ± 0.10 b	3.53 ± 0.19 a	3.51 ± 0.11 a	3.68 ± 0.06 a	0.007
Globulin	g/dL	3.52 ± 0.06 a	3.59 ± 0.19 a	3.76 ± 0.23 a	3.81 ± 0.27 a	0.194
Urea nitrogen (BUN)	mg/dL	13.23 ± 2.01 a	13.47 ± 1.64 a	14.31 ± 0.52 a	13.12 ± 0.85 a	0.632
Creatinine	mg/dL	1.10 ± 0.15 a	1.14 ± 0.03 a	1.05 ± 0.04 a	1.17 ± 0.06 a	0.254
Lactate	mmol/L	1.27 ± 0.04 a	1.05 ± 0.22 a	1.23 ± 0.14 a	1.06 ± 0.12 a	0.109
Glucose: insulin ratio	ratio	8.44 ± 0.38 a	9.09 ± 1.25 a	8.43 ± 0.57 a	9.62 ± 1.83 a	0.433

Values are the mean ± SD of the two-month average per heifer (n = 32 heifers/treatment; 128 heifers in total). Different letters in the same row indicate differences in Tukey HSD (*p* < 0.05).

**Table 3:** Reproductive hormone profile of dairy heifers.

Parameter	Unit	R1	R2	R3	R4	*p*
Estradiol-17β (E2)	pg/mL	7.02 ± 0.64 a	7.79 ± 1.58 a	6.92 ± 1.27 a	6.97 ± 0.48 a	0.651
Progesterone (P4)	ng/mL	0.57 ± 0.14 a	0.64 ± 0.10 a	0.70 ± 0.25 a	0.75 ± 0.22 a	0.584
LH	ng/mL	1.52 ± 0.31 a	1.63 ± 0.17 a	1.50 ± 0.21 a	1.62 ± 0.21 a	0.820
FSH	ng/mL	0.97 ± 0.12 a	0.99 ± 0.13 a	0.92 ± 0.08 a	0.85 ± 0.18 a	0.528
Anti-Müllerian hormone (AMH)	ng/mL	0.82 ± 0.05 a	0.96 ± 0.16 a	0.91 ± 0.16 a	0.88 ± 0.05 a	0.493
Prolactin	ng/mL	5.36 ± 0.29 a	5.93 ± 0.46 a	6.04 ± 0.77 a	6.00 ± 1.08 a	0.520
Leptin	ng/mL	2.63 ± 0.37 a	2.58 ± 0.30 a	2.73 ± 0.29 a	2.80 ± 0.38 a	0.793

Values are the mean ± SD of the two-month average per heifer (n = 32 heifers/treatment; 128 heifers in total). Different letters in the same row indicate differences in Tukey HSD (*p* < 0.05).

**Table 4:** Growth hormone–anabolic and cortisol profiles.

Parameter	Unit	R1	R2	R3	R4	*p*
Growth hormone (GH)	ng/mL	2.20 ± 0.81 a	2.09 ± 0.27 a	2.04 ± 0.21 a	2.27 ± 0.25 a	0.896
IGF-1	ng/mL	195.29 ± 18.84 a	188.48 ± 5.16 a	205.63 ± 11.29 a	199.09 ± 15.39 a	0.386
Insulin	µIU/mL	9.20 ± 1.18 a	8.43 ± 0.73 a	8.79 ± 0.70 a	9.18 ± 1.99 a	0.803
T3	ng/mL	1.36 ± 0.09 a	1.42 ± 0.05 a	1.43 ± 0.08 a	1.49 ± 0.03 a	0.107
T4	µg/mL	73.42 ± 6.04 a	73.50 ± 7.49 a	74.93 ± 5.96 a	80.19 ± 8.50 a	0.509
Cortisol	ng/mL	16.15 ± 0.65 a	15.37 ± 0.80 a	16.99 ± 2.98 a	14.67 ± 2.88 a	0.480

Values are the mean ± SD of the two-month average per heifer (n = 32 heifers/treatment; 128 heifers in total). Different letters in the same row indicate differences in Tukey HSD (*p* < 0.05).

## Data Availability

No data or results are stored in an official repository. All data and materials related to this research are presented in this article or are available upon request or through correspondence with the authors.

## Abbreviations

The following abbreviations are used in this manuscript:

MCV	Mean Corpuscular Volume
MCH	Mean Corpuscular Haemoglobin
MCHC	Mean Corpuscular Haemoglobin Concentration
RDW-CV	Red Cell Distribution Width–Coefficient of Variation
MPV	Mean Platelet Volume
NEFA	Non-Esterified Fatty Acid
HDL	High-Density Lipoprotein
LDL	Low-Density Lipoprotein
LH	Luteinizing Hormone
FSH	Follicle-Stimulating Hormone
IGF-1	Insulin-Like Growth Factor 1
T3	Triiodothyronine
T4	Thyroxine (Tetraiodothyronine)
